# Identification of Genetic Diversity, Pyrrocidine-Producing Strains and Transmission Modes of Endophytic *Sarocladium zeae* Fungi from *Zea* Crops

**DOI:** 10.3390/microorganisms10071415

**Published:** 2022-07-14

**Authors:** Qianhe Liu, Linda J. Johnson, Emma R. Applegate, Karoline Arfmann, Ruy Jauregui, Anna Larking, Wade J. Mace, Paul Maclean, Thomas Walker, Richard D. Johnson

**Affiliations:** AgResearch Limited, Grasslands Research Centre, Palmerston North 4442, New Zealand; qianhe.liu@agresearch.co.nz (Q.L.); linda.johnson@agresearch.co.nz (L.J.J.); emma.applegate@agresearch.co.nz (E.R.A.); karoline.arfmann@agresearch.co.nz (K.A.); ruy.jauregui@agresearch.co.nz (R.J.); anna.larking@agresearch.co.nz (A.L.); wade.mace@agresearch.co.nz (W.J.M.); paul.maclean@agresearch.co.nz (P.M.); thomas.walker@agresearch.co.nz (T.W.)

**Keywords:** *Sarocladium zeae*, maize endophytes, pyrrocidines, PCR assay, genotyping by sequencing, genetic diversity

## Abstract

Genotyping by sequencing (GBS) was used to reveal the inherent genetic variation within the haploid fungi *Sarocladium zeae* isolated from diverse *Zea* germplasm, including modern *Zea mays* and its wild progenitors—the teosintes. In accordance with broad host relationship parameters, GBS analysis revealed significant host lineages of *S. zeae* genetic diversity, indicating that *S. zeae* genetic variation may associate with different evolutionary histories of host species or varieties. Based on a recently identified PKS-NRPS gene responsible for pyrrocidine biosynthesis in *S. zeae* fungi, a novel PCR assay was developed to discriminate pyrrocidine-producing *S. zeae* strains. This molecular method for screening bioactive strains of *S. zeae* is complementary to other approaches, such as chemical analyses. An *e*GFP-labelled *S. zeae* strain was also developed to investigate the endophytic transmission of *S. zeae* in *Z. mays* seedlings, which has further improved our understanding of the transmission modes of *S. zeae* endophytes in maize tissues.

## 1. Introduction

In maize crops, some of the most commonly encountered fungal pathogens are *Aspergillus* and *Fusarium* spp. These fungi are known to produce mycotoxins, such as aflatoxins and fumonisins which are linked to many different health problems in humans and animals, and are known to be carcinogenic, immunotoxic, mutagenic, nephrotoxic, neurotoxic and teratogenic [1]. Mycotoxin-producing fungi not only contaminate crops in the field but the toxins they generate have been detected at appreciable levels in harvested products, threatening the safety of food for animals and humans.

*Sarocladium zeae* (synonym: *Acremonium zeae*) [2] is a protective fungal endophyte, typically producing symptomless kernel association, that acts antagonistically towards the kernel-rotting and mycotoxin-producing fungi *Aspergillus flavus* and *Fusarium verticillioides* in maize [3,4,5] and *F. graminearum* in wheat [6]. *Sarocladium zeae* is recognized as a seedborne endophyte of maize [7] and a systemic endophyte of wheat [6]. Earlier histopathological studies and fungal isolations from dissections of asymptomatic *Zea mays* kernels revealed that *S. zeae* was frequently isolated from excised embryos and endosperm [8], although further evidence is needed to confirm embryo colonisation. There have been no reports that *S. zeae* isolates from maize produce any metabolites toxic to animals or plants [5,9]. There are no records of naturally occurring *S. zeae* having been isolated from plants other than maize or sorghum [10,11] which suggests an evolutionary relationship between *S. zeae* and these genera of cereal hosts. Synthetic infections can be established in other genera such as *Triticum* [6].

*Sarocladium zeae* produces secondary metabolites—pyrrocidine A and B, which are polyketide-amino acid-derived antibiotics displaying significant antifungal activity in vitro against *A. flavus* and *F. verticillioides* as well as inhibiting the growth of *F. verticillioides* within *S. zeae* colonised kernels [4]. Pyrrocidine A also exhibits potent activity against major stalk and ear rot pathogens of maize, including *F. graminearum*, *Nigrospora oryzae*, *Stenocarpella (Diplodia) maydis* and *Rhizoctonia zeae* in addition to potent activity against *Clavibacter michiganense* subsp. *nebraskense*, the causal agent of Goss’s bacterial wilt of maize [3,4]. Most interestingly, a recent study revealed that pyrrocidines act, likely through interacting with the *Fv*ZBD1 gene, to switch off fumonisin biosynthesis in *F. verticillioides* [12]. This suggests that *S. zeae* can manipulate the secondary metabolism of a competitor fungus, thereby repressing the production of this mycotoxin, with the potential to reduce concerns for food safety [12]. Thus, bioactive *S. zeae* strains producing pyrrocidines are potentially a confounding variable in maize variety trials for resistance to pathogenic microbes and their mycotoxins [3].

Due to genetic variation, *Sarocladium zeae* (a haploid) strains differ in their ability to produce antibiotic secondary metabolites and not all *S. zeae* strains produce pyrrocidines. An evaluation of 154 *S. zeae* isolates accessioned by the USDA Agricultural Research Service Culture Collection (NRRL) and CBS Culture Collections from 1969 to 1992 revealed that the proportion of pyrrocidine-producing strains in all *S. zeae* strains isolated from various seed populations ranged from <1% to 98%, largely depending on differing climate conditions where the maize populations were grown [7]. *Sarocladium zeae* endophytes differing in their ability to produce pyrrocidines were suggested to be naturally selected by climate and distributed with the seeds of maize cultivars grown in commercial plantings. Nevertheless, we speculated that the selection of pyrrocidine-producing *S. zeae* strains may also be associated with the evolutionary history of host *Zea* species/varieties, particularly within the non-commercialised wild teosintes population. We further investigated this by performing genotyping by sequencing (GBS) [13] to reveal the inherent genetic variation within *S. zeae* strains, isolated from diverse *Zea* germplasm including modern *Z. mays* and its wild progenitors, and through the development of a rapid polymerase chain reaction (PCR)-based method screened strains of *S. zeae* for their ability to produce pyrrocidines.

Detection of pyrrocidine-producing *S. zeae* strains by conventional methods is based upon chemical analysis, which is costly, labour intensive and requires significant time to obtain a result [3,4]. To meet increasing demands for economic and timely analysis, new approaches for identifying pyrrocidine-producing *S. zeae* strains need to be explored. Most recently, biosynthetic gene clusters have been functionally characterised for pyrrocidine B [14] and identified for pyrrocidine A ([15], not released), and through whole genome-sequencing, we have also detected a PKS-NRPS (hybrid polyketide synthase-non-ribosomal peptide synthetase) gene cluster linked to pyrrocidine A biosynthesis (this study, see below). The identification of the pyrrocidine biosynthetic pathways [14,15] has provided a basis for using a molecular approach to rapidly screen pyrrocidine-producing *S. zeae* strains. This paper describes a specific, sensitive and robust PCR detection assay that was developed to preliminarily screen *S. zeae* strains for their potential to produce pyrrocidines, before further investigation with chemical analyses.

Whilst our own studies, and others [6,7,8], have documented *S. zeae* as being endophytic fungi, research into how host plants are colonised and how the endophyte is transmitted is lacking. To study the *S. zeae* transmission modes in more detail, we created a genetically modified strain expressing the green fluorescent protein (GFP) that was inoculated into maize seedlings and examined using fluorescence microscopy.

Overall, in this study, we aimed to (i) reveal the genetic variation within the *S. zeae* isolates by using GBS techniques; (ii) develop a PCR-based array to screen *S. zeae* strains that produce antibiotic pyrrocidines; and (iii) further investigate the transmission modes of *S. zeae* endophytes in maize tissues.

## 2. Materials and Methods

### 2.1. Seed Material

The maize kernels used for isolation of *S. zeae* fungal strains were imported from various germplasm collections, including The U.S. National Plant Germplasm System (NPGS, Ames, IA, USA), The International Maize and Wheat Improvement Centre (CIMMYT, El Batán, Mexico), Forage Genetics International (Davis, CA, USA) and PGG Wrightson Seeds (Christchurch, New Zealand). A total of 384 seed accessions, consisting of 275 landraces/cultivars of *Zea mays* and 109 wild teosinte progenitors (51 *Z. m. parviglumis*, 50 *Z. m. mexicana*, 5 *Z. diploperennis*, 2 *Z. perennis* and 1 *Z. m. nicarraguensis*), were screened for seed-associated endophytes. Those accessions were originally collected from 51 countries, mostly from North, Central and South America (Supplementary Table S1). For each accession, at least 5 individual kernels were subjected to endophyte screening.

### 2.2. Isolation of Fungal Endophytes

Due to the difference in germination features, two protocols were used for isolating endophytes in modern maize (*Z. mays*) and wild teosinte progenitors, respectively. For *Z. mays,* kernels (~25) were surface sterilised by soaking in 95% ethanol for 1 min, followed by rinsing in sterile H_2_O. The kernels were further sterilised in 30 mL 100% commercial Janola bleach containing 42 g/L active sodium hypochlorite (Pental Products Pty Ltd., Melbourne, Australia) for 4 min while gently shaking, followed by thorough rinsing of the kernels at least 5 times using sterile H_2_O. Surface-disinfected kernels were germinated on sterile 2% water agar plates (12 cm × 12 cm; 5 seeds per plate) in the dark at 23 °C for 4–7 days. Germinated seedlings free of microbial growth were aseptically dissected into ~2–3 mm sections. The sections were placed on PDA (potato dextrose agar, Oxoid Ltd., Basingstoke, UK) plates and incubated at 23 °C in a growth room and assessed for the emergence of fungal endophytes every day. For teosintes, the kernels were first surface sterilised with 12% Janola bleach for 20 min (vortexed frequently), then rinsed in sterile H_2_O. The bleach-treated kernels were sown in sterile vermiculite (in 50 mL falcon tubes) for germination. Post germination (shoot length > 3 cm), seedlings were harvested and surface sterilised again using 12% Janola bleach for 2–3 min, followed by 70% ethanol for ~30 s and thoroughly rinsed with sterile H_2_O. The treated seedlings were aseptically dissected into ~2–3 mm sections for endophyte isolation as described above.

Fungi emerging from tissue sections were isolated and purified through hyphal tip sub-culture on PDA plates. Fresh mycelia were harvested after 2 weeks and stored in 20% glycerol solution at −80 °C for long-term storage. Pure cultures on PDA plates were used to produce inoculum for experiments after identification. Notably, due to the concerns of intellectual property, we are not able to share those *S. zeae* collections. This limitation also applies to our genome sequencing data and GBS dataset as stated below.

### 2.3. Isolate Identification

Morphological characteristics were examined under 100-400x magnification using a compound microscope (BX53, Olympus, Tokyo, Japan). Genomic DNA of the fungus was extracted using the Quick-DNA^TM^ Fungal/Bacterial Miniprep Kit (Zymo Research, Irvine, CA, USA) and the ITS gene was amplified using universal primers ITS1 (5′-TCCGTAGGTGAACCTGCGG-3′) and ITS4 (5′-TCCTCCGCTTATTGATATGC-3′) following the instructions of the SapphireAmp^®^ Fast PCR Master Mix Kit (Takara Bio USA Inc., San Jose, CA, USA). The ITS amplicon was further purified (DNA Clean & Concentrator^TM^-5, Zymo Research, Irvine, CA, USA) and sequenced (Massey Genome Service, Palmerston North, New Zealand). The ITS gene sequences of *S. zeae* strains were blasted (BlastN) against the NCBI GenBank NR database and phylogenetically analysed with the top GenBank hits using Geneious R10 software (Biomatters Ltd., Auckland, New Zealand) with the neighbour-joining method (bootstrap = 1000) to determine their relatedness.

### 2.4. Whole Genome Sequencing and AntiSMASH Analysis of S. zeae Strain PI159-1.1

*Sarocladium zeae* strain PI159-1.1, a strain producing pyrrocidine A, was isolated in this study from a landrace of *Z. mays*, collected in Thailand by USA-NPGS. Fungal mycelia of the strain, grown on sterile cellophane film overlaid on PDA medium for ~2 weeks, were collected and ground in liquid nitrogen. High-quality genomic DNA was extracted according to the manual of the Quick-DNA^TM^ Fungal/Bacterial Midiprep Kit (Zymo Research, Irvine, CA, USA) and sent to BGI Tech Solution (Hong Kong) Co. Limited for de novo sequencing using the platform of PacBio Sequel with 20k library. Genome assembly was performed using the program Canu version 1.6, with an estimated genome size parameter of 40 Mb. All other parameters were left with default values, and the command was modified to skip the Gnuplot test (gnuplotTested = true) and to run on a single computer (useGrid = false). The taxonomy affiliation of the assembly was verified by blasting it against the NCBI NR database.

The gene clusters associated with secondary metabolites were predicted by using antiSMASH (Fungi version 5.1 [16]), with the criterion of 80% shared gene content to identify similar gene clusters. The sequences of gene clusters that are responsible for producing pyrrocidine metabolites in PI159-1.1 were compared with the biosynthetic gene cluster MW690134, a gene cluster of *S. zeae* strain NRRL13540 that produces pyrrocidine B and has recently been functionally characterised by Ohashi et al. [14].

### 2.5. Profiling Genetic Variation of S. zeae Isolates with GBS

Eighty-seven DNA samples of *S. zeae*, isolated from diverse hosts including various wild teosintes, landraces and cultivated varieties, were sequenced using GBS. Fungal growth and extraction of high-quality gDNA were performed as described above for whole-genome sequencing. DNA quality was checked via visualisation on ethidium bromide stained 0.8% agarose/TAE gels and then quantified using an Invitrogen Qubit 4 Fluorometer (Thermo Fisher Scientific, Waltham, MA, USA). DNA concentrations were normalised to 20 ng per μL and subsequently used for GBS library preparation.

A GBS library was generated following the methodology of Faville et al. [17], with 100 ng of DNA digested using restriction enzyme PstI (New England Biolabs, Ipswich, MA, USA) and ligated to a unique barcoded adapter and a common adapter (20 ng, pre-determined by a titration) [17]. The library was developed in 96-plex which includes a blank and samples, with samples AM56-4.F1N and PI147-2.1 in quadruplicate and sample AM13-2.1 in triplicate. The library was sequenced on one lane of an Illumina HiSeq 2500 (Illumina, San Diego, CA, USA) at AgResearch Invermay, New Zealand. This generated a library containing 1.2 M single reads of 100 nt. Initial quality control and trimming was performed using Trimmomatic [18]. The GBS reads were analysed using Tassel 5, version 5.2.40 [19] and the assembled genome as a reference with standard parameters, except that the minimum minor allele frequency was set to 0.001. The vcf table produced by this pipeline was used for further downstream analysis. The vcf table was then subject to filtering using a custom Ruby script, where SNPs (single nucleotide polymorphism) with scores less than 100, multi-allelic SNPs, SNPs with less than 10× coverage in total and indels were removed. Diagnostic plots, particularly minimum allele frequencies and SNP depth frequencies (supplementary Figures S1 and S2, respectively) were generated using the KGD package [20]. To classify isolates based on SNP similarity, the SNPs were converted into genotype calls where 0 represented homozygous reference calls and 1 represented homozygous alternative calls, and SNPs with heterozygosity were removed. A Euclidean distance matrix was generated from the genotype calls and a dendrogram of the isolates from different locations and different hosts was created using a hierarchical clustering method with complete linkage.

### 2.6. Detection of Pyrrocidine-Producing S. zeae Strains by PCR Assay

Purified *S. zeae* isolates were grown on PDA plates for 2 weeks. Mycelia were harvested and genomic DNA of 160 *S. zeae* isolates was extracted using the Quick-DNA^TM^ Fungal/Bacterial Miniprep Kit (Zymo Research, Irvine, CA, USA) according to the kit instructions. PCR detection assays for pyrrocidine-producing *S. zeae* strains were carried out using two sets of primers: PYD-1 (forward: 5′-CTCTCTATGTCGGCTCTATCAA-3′; reverse: 5′- GGTGAAGTAGCCTCTGTAGTAG -3′; amplicon = 992 bp) and PYD-2 (forward: 5′-AACCTGCGCGTTGAGTTCTA-3′; reverse: 5′-CGATGGAGTATGGTCCTGCC-3′; amplicon = 481 bp). These two sets of primers were both designed from a shared open reading frame (ORF) region of the key *pydA* gene (i.e., the PKS-NRPS gene) in both pyrrocidine A and B biosynthetic gene clusters. PYD-1 primers were employed to preliminarily distinguish pyrrocidine-producing strains from non-producing *S. zeae* isolates. The PYD-2 primers were used to further clarify these PCR results. PCR detection arrays were conducted in a T100^TM^ Thermal Cycler (BIO-RAD Laboratories, Singapore) following the instructions of SapphireAmp^®^ Fast PCR Master Mix kit (Takara Bio USA Inc., San Jose, CA, USA); and were programmed for 1 cycle of 3 min at 94 °C, 35 cycles of 5 s 98 °C (denaturalization), 5 s at 60 °C for PYD-1 or 58 °C for PYD-2 (annealing), 15 s at 72 °C (extension), and finally 1 cycle of 5 min at 72 °C. Amplification reaction was carried out in volumes of 25 µL containing ~15–30 ng of template gDNA in 1 µL, 0.5 µL of each primer (10 µM), 12.5 µL of SapphireAmp^®^ Fast PCR Master Mix. PCR products were detected in 1.5% agarose ethidium bromide gels (Life Technologies Inc., Gaithersburg, MD, USA). The GeneRuler 1kb DNA ladder (Thermo Fisher Scientific, Vilnius, Lithuania) was used as a molecular size marker.

### 2.7. Validation of Pyrrocidine-Producing S. zeae Strains by Chemical Analysis

Eleven *S. zeae* strains with different results based on the above PCR assays were selected for chemical analysis of pyrrocidines. Fungal inoculation/fermentation procedures were carried out as described by Wicklow et al. [5]. Generally, maize kernels (50 g each) were weighed into 500 mL flasks and soaked in 50 mL H_2_O overnight. Kernels were then autoclaved for 60 min and cooled down to room temperature. The autoclaved kernels were inoculated with 3 mL *S. zeae* conidial spore suspension (containing 2 × 10^4^ conidia per mL in sterile H_2_O) and fermented at 23 °C for 20 days.

Post-fermentation, infected kernels were freeze-dried, and subsamples (~1000 mg) were ground with a bead ruptor (6.4 mm zirconium bead, 4 m/s for 30 s) (FastPrep-24, M.P. Biomedicals, Irvine, CA, USA) before being extracted with 10 mL ethyl acetate (Sigma-Aldrich, St. Louis, MO, USA) by end-over-end rotation in the dark (1 h at 30 rpm). After centrifuging (4000× *g*, 2 min) (Heraeus Megafuge 16, Thermo Scientific, Osterode, Germany), the supernatant was transferred into glass screwcap Kimax tubes (13 mm × 100 mm) and dried under a stream of nitrogen. The residue was resuspended in 2 mL of hexane (Sigma-Aldrich, St. Louis, MO, USA) and transferred to a second glass screwcap Kimax tube (13 mm × 100 mm). Partition with 3 × 2 mL of acetonitrile (Sigma-Aldrich, St. Louis, MO, USA) (rinsing the original Kimax tube), transferring the acetonitrile portion into a fresh screwcap Kimax tube (13 mm × 100 mm). The combined acetonitrile portions were dried under a stream of nitrogen, then resuspended in 1 mL ethyl acetate and transferred to a 2 mL glass HPLC vial. The ethyl acetate was removed under a stream of nitrogen, then the residue dissolved in 200 µL of 50% acetonitrile, and then analysed by LC-HRMS. Compounds were separated using a Polar-RP column (100 × 2.0 mm, 2.5 µm) (Phenomenex, Torrance, CA, USA) using a water/methanol gradient (0.1% acetic acid buffered). Peaks were detected using a Q-Exactive (Thermo Fisher Scientific, Waltham, MA USA) collecting MS^1^ spectra from 200–500 *m/z* and selected PRMs for predicted metabolites.

### 2.8. Investigation of S. zeae Fungal Transmission Modes

The endophytic characterisations of *S. zeae* fungi that were inoculated into maize seedlings were investigated through genetically modified *S. zeae* strain PI159-1.1-*e*GFP*#13*, which produces green fluorescence and is helpful for microscopic observation. This strain was generated from PI159-1.1 by randomly cloning a gene coding elected green fluorescence protein (*e*GFP) into the PI159-1.1 fungal genome, via an *e*GFP-constructed vector (pTefp119-*e*GFP-HygR). Protoplast preparation and *e*GFP transformation followed the modified protocols described by Vollmer and Yanofsky [21] and Oliver et al. [22] as used by Johnson et al. [23], respectively.

Seeds of the modern *Z. mays* cultivar N101 (from PGG Wrightson Seeds, NZ)) and fast-flowering mini-maize (FFMM), which was kindly provided by Professor James Birchler via Maize Genetics Coop Stock Center (Urbana, IL, USA), were artificially inoculated with *S. zeae* strain PI159-1.1-*e*GFP#13, by immersing the surface-sterilised kernels into fungal conidia suspension (2 × 10^6^ conidia per mL in 0.05% Tween-20 solution) for 1 h. In order to avoid the genetically modified *S. zeae* releasing into the environment, *S. zeae*-inoculated N101 or FFMM seeds were sowed and grown either in sealed transparent pots (8.5 cm diameter and 24 cm height) for 3 weeks in a growth room or in sealed Vented Large Propagator (58 cm length × 38 cm width × 84 cm height, Egmont Seed Company, New Plymouth, New Zealand) for 3 months in a growth chamber, respectively. For both trials, pre-autoclaved soil plus potting mix (2:1 *v*/*v*) were supplied as growth media; and the growth regime was set up at 25 °C/14 h light and 20 °C/10 h dark. Colonisation and endophytic characterisation of the *e*GFP-labelled *S. zeae* strain were microscopically investigated by using an Olympus Fluoview FV10i confocal laser scanning microscope (Tokyo, Japan) and an Olympus BX63 automated fluorescence microscope (Tokyo, Japan). Notably, the mini maize plants growing in the sealed propagator did not complete their full lifecycle, even though the trial has been extended to 3 months, and the plants died at the flowering stage.

Due to the failure to produce seed from the mini maize plants that have been inoculated with *e*GFP-labelled *S. zeae*, multiple kernels (>10) of imported *Z. mays* that have naturally colonised with *S. zeae* fungi were selected for investigating colonisation sites of *S. zeae* fungi in seeds. Seed embryos were dissected out from symptomless and surface-disinfected kernels, and these embryos and the remaining seed residues were plated on PDA to check for the emergence sites of *S. zeae*.

## 3. Results

### 3.1. Determination of Pyrrocidine Biosynthetic Pathway in S. zeae Strain PI159-1.1

The *S. zeae* strain PI159-1.1 is a pyrrocidine A-producing isolate (see section below). A high-quality draft genome (31.8 Mb) of this strain with a G+C content of 52.8% was obtained by de novo genome sequencing using the BGI PacBio Sequel platform. The in-silico analysis using antiSMASH (Fungal version 5.1.0 [16]) predicted the presence of 3 PKS-NRPS and 1 PKS-NRPS-like gene clusters that are probably associated with secondary metabolite biosynthesis. Amongst these, one of them showed significant similarity with the functionally characterised pyrrocidine B biosynthetic gene cluster (MW690134.1) in *S. zeae* strain NRRL13540 [14]. Nucleotide sequences of this whole PKS-NRPS gene cluster in PI159-1.1 share 94.2% similarity with the reported MW690134.1 gene cluster in strain NRRL13540. All gene elements, as described by Ohashi et al. [14] in cluster MW690134.1, were also found in the PKS-NRPS cluster of strain PI159-1.1, indicating that the synthesis of pyrrocidine A and pyrrocidine B share the same biosynthetic pathway. Within the gene cluster, the amino acid sequence of the key enzyme PydA (4194aa) showed a similarity of 98.8% between the two strains. However, compared with strain NRRL13540 [14] an additional sequence fragment (~1530 bp) is present in strain PI159-1.1, located between genes of *pydG* and *pydF*.

### 3.2. GBS Analysis of S. zeae Isolates to Determine Genetic Diversity

**Figure 1:**
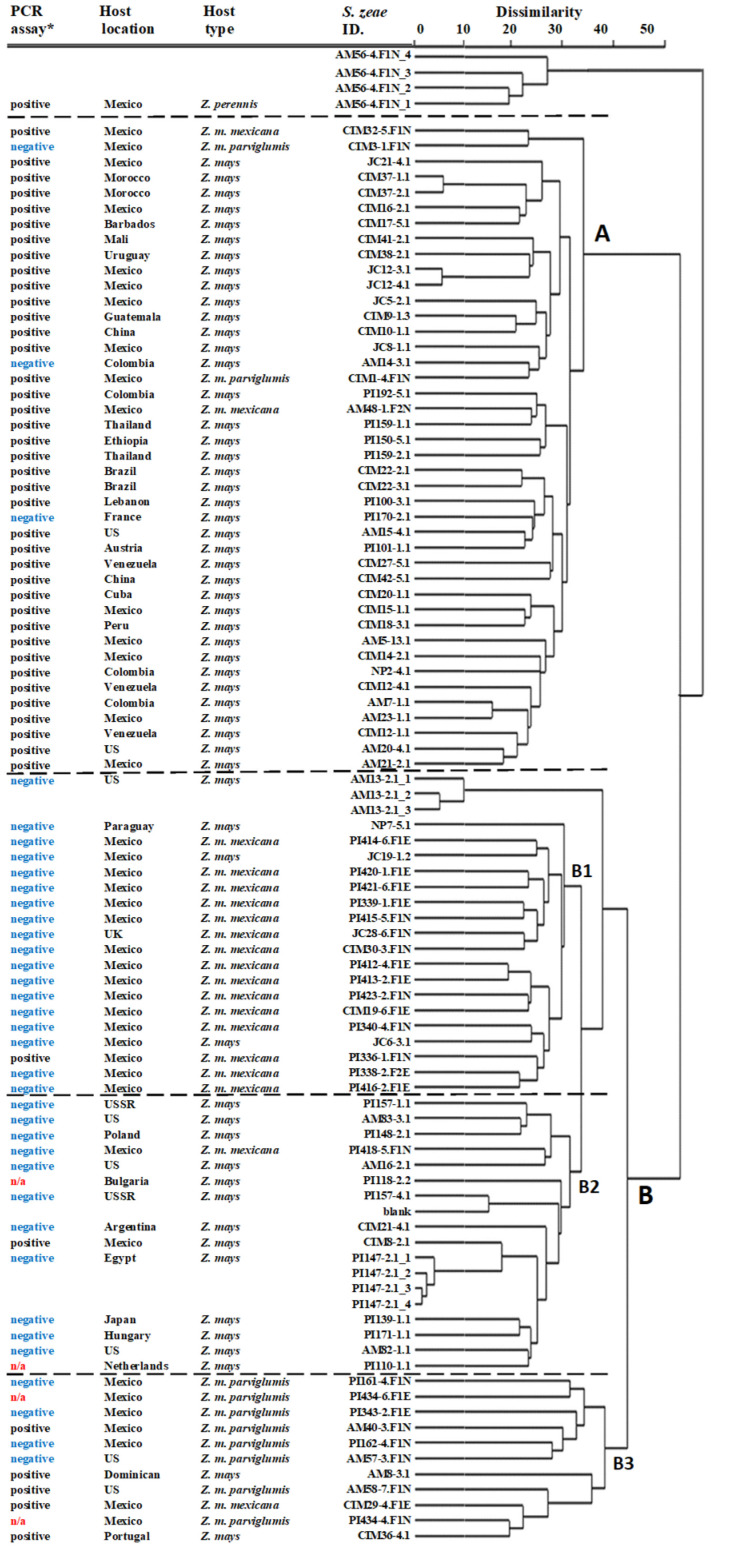
Dendrogram of a GBS analysis for 87 strains of *Sarocladium zeae.*: PCR assay: positive—*pydA* gene detected; negative—*pydA* undetected; n/a—not analysed. *PCR assay was conducted to detect the key *pydA* gene that associates with pyrrocidines production. Different *S. zeae* groups separated by horizontal dashed lines. A—group dominated by *pydA* positive strains; B—group dominated by *pydA* negative strains; B1—subgroup dominated by strains isolated from *Z. m. mexicana*; B2—subgroup dominated by strains isolated from *Z. mays*; B3—subgroup dominated by strains isolated from *Z. m. parviglumis.* GBS analysis for AM56-4.F1N and PI147-2.1 was quadruplicated and AM13-2.1 triplicated.

GBS allows genetic variation to be resolved, in this case by using SNPs. To classify isolates based on SNP similarity, a dendrogram of 87 strains from diverse hosts was created using a hierarchical clustering method with the Euclidean distance method (Figure 1). The host species appeared to influence *S. zeae* genetic diversity significantly, with most *S. zeae* strains isolated from *Z. mays* clustering in Group-A (91%) and Group-B2 (93%); strains isolated from *Z. m. mexicana* clustered in Group-B1 (83%), and strains from *Z. m. parviglumis* in Group-B3 (82%) (Figure 1). Results indicate that the genetic diversity of *S. zeae* isolates is linked to the host species/variety that they were isolated from, particularly apparent when comparing isolates from modern *Z. mays* and those from the wild teosintes. The genetic variation of *S. zeae* isolates also varied within *Z. mays* species in Group-A and Group-B2. There was no obvious trend of genetic variation associated with host geographical locations.

In addition to host lineages, interestingly, the genetic variation of *S. zeae* appeared to be linked to the pyrrocidine biosynthetic *pydA* gene as well. The fungal genetic variation detected by GBS was mirrored by the presence of *pydA*. As shown in Figure 1, most of the PCR-positive *S. zeae* isolates (93%) were clustered in *Z. mays* dominated by Group-A, while PCR-negative isolates were clustered in Group-B1 (94%; *Z. m. mexicana* as predominant hosts) and Group-B2 (92%; *Z. mays* as predominant hosts).

### 3.3. Development of PCR Assay for Simple and Rapid Screening of Pyrrocidine-Producing S. zeae Strains

**Figure 2:**
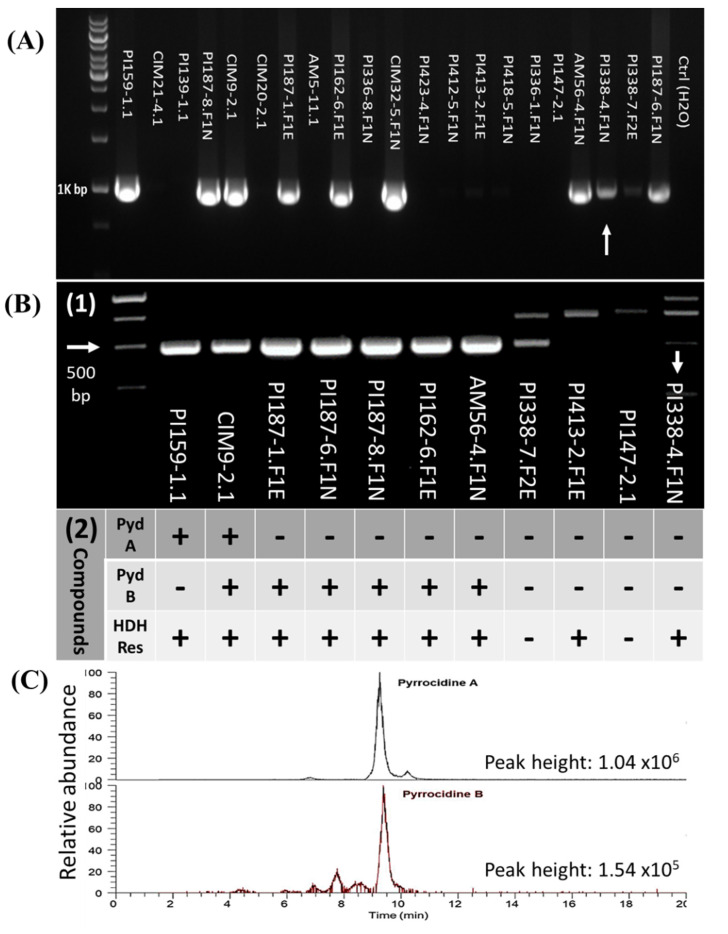
PCR detection assay of pyrrocidine-producing *Sarocladium zeae* strains and chemical analysis of pyrrocidine-associated compounds. (**A**) PCR assay by PYD-1 primers; (**B**) PCR assay by PYD-2 primers (1) and chemically detected compounds (2). Pyd-A—pyrrocidine A; Pyd-B—pyrrocidine B; HDH-Res—hydroxydihydroresorcylide. (**C**) Relative abundance of pyrrocidine A and B in *S. zeae* strain CIM9-2.1.

In the present study, a total of 206 *S. zeae* isolates were isolated from different *Zea* accessions collected from different geographical locations (Table 1). Due to multiple *S. zeae* isolates recovered from a single seed accession, 160 *S. zeae* isolates (83 from landraces and 77 from teosintes, Table 2) were subjected to a PCR assay detecting the *pydA* gene, screening strains for production of pyrrocidines. DNAs extracted from pure mycelia of the 160 isolates were screened for pyrrocidine production by PCR using the PYD-1 primers [15]. *Sarocladium zeae* isolates were clearly distinguished by PYD-1 primers, with a single amplicon of 992 bp associated with strains putatively possessing the pyrrocidine biosynthetic gene *pydA*, and the absence of amplified products for others (Figure 2A). In some rare cases, mostly occurring in the strains isolated from teosintes, the PYD-1 primers produced a faint band for strains that did not possess *pydA* (arrowed in Figure 2A) which made the test ambiguous. To resolve this, we designed an additional primer set (PYD-2) that provided complementary to PYD-1 for reducing this ambiguity. For *pydA* positive strains, the PYD-2 primers generated a clear single band, as opposed to the multiple off-specific bands produced in *pydA* negative strains (Figure 2(B1)). In combination, these two primer sets were able to identify *pydA* positive strains, which produced a single, clear band with both PYD-1 and PYD-2 primer sets (at 992bp and 481bp, respectively). Notably, the PYD-2 primer set was not intended to be used alone, but to filter out the small number of *pydA* negative isolates that falsely test positive with PYD-1 primers.

For validating the PCR assays, 11 *S. zeae* isolates with different results based on the PCR assays were selected and subjected to chemical analysis for pyrrocidine metabolites (Figure 2(B2)). Of these 11 isolates, both pyrrocidine A and B were detected in strain CIM9-2.1 (Figure 2C), pyrrocidine A in strain PI159-1.1, and pyrrocidine B in the 5 other strains. All these pyrrocidine-producing strains corresponded to a positive PCR assay. There were no pyrrocidines detected in any of the 4 isolates that showed a negative PCR assay, including the isolate PI338-4.F1N in which there was a faint band amplified by PYD-1 primers. Results from chemical analysis confirmed the PCR detection assay.

### 3.4. Transmission Modes of Endophytic S. zeae in Maize

**Figure 3:**
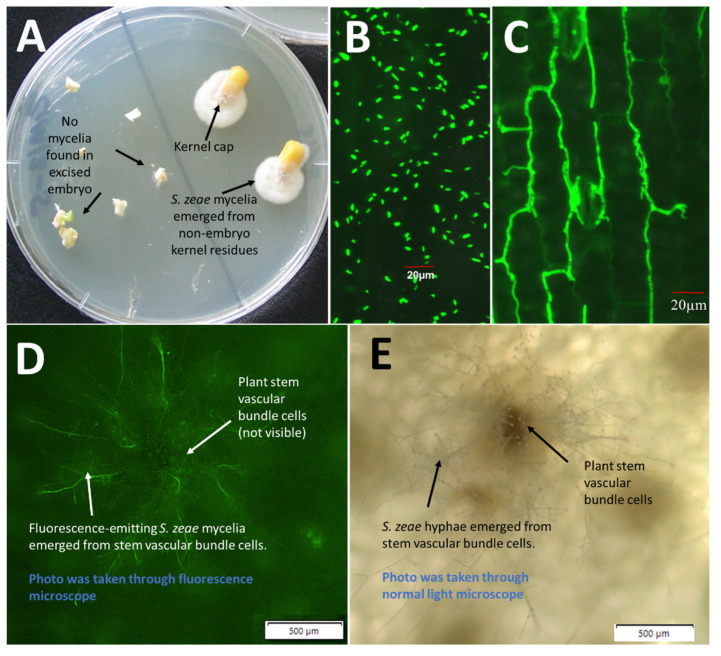
Endophytic colonisation of *Sarocladium zeae* mycelia in maize tissues. (**A**) Mycelia of *S. zeae* were associated with kernel caps, rather than inhabiting seed embryos (arrowed). *S. zeae*-colonised kernels were surface sterilised, and embryos were separated from other seed tissues and plated on PDA plates for mycelia to emerge. (**B**) Fluorescence-emitting *S. zeae* conidia repropagated on leaf blade. These conidia were reproduced in situ by plant-colonised *S. zeae* which were initially inoculated through seed. (**C**) Leaf blade endophytically colonised by fluorescence-emitting *S. zeae* mycelia. In panel B and C, seedlings were initially inoculated with the *e*GFP-labelled *S. zeae* strain at seed stage and grew in sealed pots for 3 weeks. Photos were taken through a confocal laser scanning microscope. (**D**) Fluorescence-emitting *S. zeae* mycelia emerged from stem vascular bundle cells of a 3-month-old mini maize plant. The plant was initially inoculated with *e*GFP-labelled *S. zeae* strain at seed stage. Photo was taken through a fluorescence microscope. (**E**) Visible plant stem vascular bundle cells and *S. zeae* mycelia under normal light. Photo taken through a normal light compound microscope.

Despite lacking evidence, seedborne *S. zeae* endophytes in maize were often speculated to inhabit the seed embryo and to transmit vertically through vascular tissues. In this study, however, no *S. zeae* hyphae emerged from the dissected embryo specimens (Figure 3A). Instead, *S. zeae* mycelia were recovered from the kernel caps, originating from the pedicel and abscission layer, indicating that *S. zeae* may not infect the embryo and endosperm naturally.

Confocal microscopic examination revealed that repropagated *S. zeae* conidia (Figure 3B) were spread on the leaf blade of a 3-week-old *Z. mays* seedling, which was pre-inoculated with an *e*GFP-labelled *S. zeae* strain through seed. Conidia-originating *S. zeae* mycelia that colonised the intercellular spaces of the leaf blade were also revealed (Figure 3C). In addition, microscopic examination clearly revealed that endophytic mycelia of *S. zeae* grew within internal stem vascular bundle cells of an *e*GFP-labelled *S. zeae*-colonised mini maize (Figure 3D,E), indicating an important transmitting approach keeping *S. zeae* vertically transmitted into next-generation seeds of maize.

## 4. Discussion

*Sarocladium zeae* is characterized as a “protective fungal endophyte” of maize. The potent antifungal and antibacterial properties of pyrrocidines produced by *S. zeae* were first described by Wicklow and Poling [4] and He et al. [24], respectively. Recently, pyrrocidine has been demonstrated to switch off fumonisin biosynthesis in *F. verticillioides* [12], a major mycotoxigenic food safety threat. All these studies suggest that *S. zeae* is a potential confounding variable in maize variety trials for resistance to pathogenic microbes and their mycotoxins [7]. In the USA, *Sarocladium zeae* has been used to effectively eliminate mycotoxin accumulation in the kernels caused by *F. verticillioides* and *A. flavus* that may produce fumonisin and aflatoxin [1]. *Sarocladium zeae* is not reported to synthesize secondary metabolites harmful to plants, nor does it cause any ear or stem rot symptoms [5,12].

Bioactive *S. zeae* strains produce pyrrocidine A and B, with pyrrocidine A being more active than B [5]. Pyrrocidine A differs from pyrrocidine B by the presence of a double bond in its lactam ring resulting, by an unknown mechanism, in a higher toxicity of pyrrocidine A against a number of fungal and bacterial species, compared to pyrrocidine B [4,5,12]. As demonstrated in strain CIM9-2.1 in this study, pyrrocidine A and B can both be detected in *S. zeae*-infected kernels. Biosynthesis of pyrrocidine A and pyrrocidine B appeared to share the same biosynthetic pathway. When sequences of pyrrocidine biosynthetic gene clusters (BGC) were compared between the *S. zeae* strains producing various pyrrocidines, the nucleotide sequences of strain PI159-1.1 (pyrrocidine A producing; this study) showed a 94.4% similarity with the sequences in strain NRRL13540 (pyrrocidine B producing [14]). The translated amino acid sequences of a key PKS-NRPS gene (*pydA*) in the pyrrocidine BGC shared a similarity of 98.8% between the two pyrricidine A- and B-producing strains. The *pydA* gene has been suggested to be responsible for the biosynthesis of the lactam ring (the tricarbocyclic core) in most pyrrocidine-like compounds [25]. Since the pyrrocidine biosynthetic gene cluster in strain NRRL13540 has recently been functionally characterised [14], we believe that this improved knowledge on the biosynthetic pathways of pyrrocidines, particularly the *S. zeae* specific *pydA* gene, may provide a foundation for, and facilitate the adaptation of, molecular genetic techniques for screening pyrrocidine-producing *S. zeae* strains.

In *Zea* crops, there is strong selection for pyrrocidine production among *S. zeae* fungal populations [7]. It is therefore important to discriminate pyrrocidine-producing strains from non-pyrrocidine-producing strains for studying the biosynthesis of pyrrocidines, as well as gaining a detailed knowledge of the potential bioactivity of various fungal strains. Current methods for identifying pyrrocidine-producing *S. zeae* strains are costly and often involve labour- and time-consuming tasks that require expertise in chemical analysis. To meet increasing demands for accurate, economic, and timely analysis, new tools for identifying pyrrocidine-producing *S. zeae* strains must be explored. Nucleic acid-based identification systems have the advantage of differentiating strains based on their genetic information. In this work, PCR primers were designed from *pydA* gene with shared sequences for both pyrrocidine A- and B-producing *S. zeae* strains; and PCR detection assays were developed for the first time to identify both pyrrocidine A- and B-producing *S. zeae* strains. The PCR detection assays provide a simple and rapid method for preliminarily screening pyrrocidine-producing *S. zeae* strains. As mentioned above, the PYD-2 primer set was not intended to be used alone, but to filter out the small number of *pydA* negative isolates that falsely test positive with PYD-1 primers.

In this work, the PCR-positive *S. zeae* strains were mostly associated with well-clustered *Z. mays* host Group-A, while PCR negative *S. zeae* strains were commonly associated with teosintes Group-B1 (dominated by *Z. m. mexicana*), although within-species variation also existed in *Z. mays* (between Group-A and Group-B2). The results clearly reflected fungal genetic diversity, as analysed by the GBS. The mechanisms involved in the gene loss or gain in the pyrrocidine biosynthetic gene cluster is unclear. Wicklow et al. [7] suggested that natural environment, such as different climate conditions, may impact the selection of *S. zeae* strains producing pyrrocidines. In regions where maize is particularly vulnerable to pathogen attack following the damaging effects of drought and temperature stress, selection may favour *S. zeae* endophytes that produce pyrrocidines as acquired chemical defences [7]. This kind of stress-induced selection is highly possible and has been observed in other fungal species, e.g., heat stress-induced different gene expression in mycorrhizal *Tuber borchii* [26]. Nevertheless, our results, in terms of the complemented profiles between PCR assay and GBS analysis, strongly suggest that host species/varieties may play a more important role in influencing *S. zeae* genetic diversity and selection of pyrrocidine-producing strains. We therefore conclude that, in addition to the natural selection as described above, pyrrocidine biosynthesis in *S. zeae* may have different evolutionary histories, possibly associated with host domestication from wild progenitors of modern maize. The observation that a large majority of *S. zeae* isolates from wild teosintes do not produce pyrrocidines invites further consideration of endophyte contributions to maize defence from pathogen attack. The findings from this study are expected to help develop effective strategies for the management of biocontrol endophytes in maize.

In this work, a total of 206 *S. zeae* fungi have been isolated from diverse maize germplasm, including 275 landraces/cultivars and 109 wild teosinte progenitors of *Zea* crops collected from 51 countries geographically. Those *S. zeae* fungi were more commonly discovered from accessions collected from North, Central and South America than other regions/countries. There were no *S. zeae* fungi isolated from New Zealand-produced *Z. mays* seeds although more than 46 accessions have been screened. In some seed accessions, multiple *S. zeae* isolates were recovered from a single accession, but usually isolated from different kernels. Those multiple strains mostly shared a similar genetic background in terms of the *pydA* gene.

Studies via traditional morphological description and ITS amplicon sequencing often revealed no obvious variation in *S. zeae* isolates. This study identified genetic diversity within *S. zeae* for the first time using GBS technology where SNP detection and genotyping are performed simultaneously [13,17]. GBS analysis clustered the *S. zeae* fungal isolates into clearly host-related groups, demonstrating significant host lineages of *S. zeae* genetic diversity. Host lineages of genetic variation have been reported in other microorganisms such as in *Pseudoperonospora cubensis* [27]. Our observation of the host-linked genetic variation of *S. zeae* provided the first view of the *S. zeae* genetic diversity at the strain level, in accordance with broad host relationship parameters. We speculated that the genetic diversity of *S. zeae* was likely to be linked to host evolution from ancestor teosintes to modern maize.

*S. zeae* is the most prevalent seedborne fungus associated with maize seed, reportedly as high as 39.5% of the seeds [7,28]. This is consistent with our observation that *S. zeae* isolates make up ~40% of our whole fungal collection in the present study. Seedborne *S. zeae* endophytes in maize were often thought to inhabit the seed embryo and transmit through vascular tissues. This observation of *S. zeae* residing in the embryo can be dated back to early histopathological studies on “sound-appearing” maize kernels, which revealed that *S. zeae* fungi were often detected in the embryo and endosperm [5,8]. It was speculated that *S. zeae* fungi are able to vertically transmit via seed and propagate in the next generation of its host, such as the well-studied *Epichloë* endophytes [29]. Since then, however, there has been no further experimental evidence to support the finding of *S. zeae* being embryo associated. In the present study, no *S. zeae* fungi were recovered from the dissected seed embryos but mycelia were recovered from the pedicel and abscission layer of the seed, suggesting that *S. zeae* more likely inhabits the pedicel and abscission layer of asymptomatic seeds, rather than the seed embryo [5,8].

*S. zeae* was also reported to be the predominant fungus recovered from stem core tissues of naturally infected healthy maize plants [8,30]. An earlier study by Reddy and Holbert [31] proposed that *S. zeae* is a systemic seedborne fungus of maize that infects the plant through the vascular system, eventually invading the ears and seeds. These observations are consistent with our observation of *e*GFP-labelled *S. zeae*, clearly showing that *S. zeae* can be endophytically transmitted within stem vascular cells. By using scanning electron microscopy, Kemp et al. [6] demonstrated that *S. zeae* spores can be transmitted vertically to the progeny of infected wheat plants, suggesting the possibility that conidia, potentially including the inoculum itself, may be transported within vascular tissue even in the absence of active growth. The authors considered that this type of passive dispersal may explain why recovery of *S. zeae* from wheat tissue pieces was sometimes discontinuous; conidia may be carried variable distances before becoming lodged or otherwise being obstructed from continuing with the flow of the transpiration stream [6].

Regarding the study of *S. zeae* transmission in maize, earlier investigation mostly focused on the transmitting modes in non-foliar tissues, such as in seeds and stems. Comparably, less attention was paid to *S. zeae* colonisation on leaves via conidia. *Sarocladium zeae* are a conidia-rich species. As illustrated in Figure 3B, conidia repropagated by *S. zeae* were often observed on intact leaves of maize seedlings. By using an *e*GFP-labelled *S. zeae* strain, the confocal laser scanning microscopy clearly revealed that conidia-germinated mycelia of the fungus can transmit and spread within intercellular spaces of the leaf blade, suggesting that *S. zeae* colonisation via propagated conidia may be another important transmission strategy of the endophyte, particularly in the tissue of leaf blades. Conidia of *S. zeae* are very small compared to most other fungi, at approximately 3.5 to 6 µm in length and 1.2 to 1.8 µm in width [6,32]. This suggests the possibility that conidia can penetrate through leaf stomata and grow into leaf intercellular tissues.

In summary, the present study provides further evidence that the effectiveness of biological control of *S. zeae* species varies among strains, which motivates the continued screening of additional strains. The findings of the present study provide preliminary information on the adaptation of molecular techniques for screening *S. zeae* strains producing pyrrocidines and significantly expands our knowledge of the genetic variation within this fungal species at the strain level. Our study also demonstrated that *S. zeae* endophytes can be vertically transmitted through stem tissues into next-generation seeds where they may inhabit seed caps rather than embryos. Endophytic colonisation of conidia-germinated mycelia of *S. zeae* in maize leaf blades may provide another important transmission strategy of the endophyte fungi.

## Figures and Tables

**Table 1:** *Sarocladium zeae* isolated from *Zea* crops.

Host	*S. zeae*-Infected Accessions	*S. zeae* Isolates
Total	131	206
Landraces (*Zea mays*)	60	87
Teosintes total	71	119
*Z. diploperennis*	3	6
*Z. m. mexicana*	30	49
*Z. m. parviglumis*	37	62
*Z. perennis*	1	2

**Table 2:** PCR screening for *pydA* gene among *Sarocladium zeae* isolates. The number of positive isolates out of the total number screened are shown for different source accession types within the *Zea* genus. Multiple *S. zeae* were isolated from some accessions.

Host	*S. zeae*-Infected Accessions ^1^	*S. zeae* Isolates ^2^
Total	101	160
Landraces (*Z. m. mays*)	33/49	60/83
Teosintes total	19/52	21/77
*Z. diploperennis*	3/3	5/5
*Z. m. mexicana*	4/25	4/40
*Z. m. parviglumis*	11/23	11/31
*Z. perennis*	1/1	1/1

^1^ The number of accessions with *pydA* detection. ^2^ Total number of *S. zeae* isolates from different accessions.

## Supplementary Materials

The following supporting information can be downloaded at: https://www.mdpi.com/article/10.3390/microorganisms10071415/s1, Figure S1: Histogram of MAF; Figure S2: Histogram of mean SNP depth; Table S1: *Zea* seed accessions screened in this study.
