# Supplementary material for: Identification of Genetic Diversity, Pyrrocidine-Producing Strains and Transmission Modes of Endophytic Sarocladium zeae Fungi from Zea Crops
Source: Microorganisms. 2022 Jul 14;10(7):1415. doi: 10.3390/microorganisms10071415 (PMC9316807; doi:10.3390/microorganisms10071415)
Supplement: Supplementary file 1 [file microorganisms-10-01415-s001.zip › microorganisms-1788420-supplementary.pdf]

Supplementary Materials

Identification of Genetic Diversity, Pyrrocidine-Producing Strains and Transmission Modes of Endophytic *Sarocladium zeae* Fungi from *Zea* Crops

Qianhe Liu, Linda J. Johnson, Emma R. Applegate, Karoline Arfmann, Ruy Jauregui, Anna Larking, Wade J. Mace, Paul Maclean, Thomas Walker, Richard D. Johnson*

AgResearch Limited, Grasslands Research Centre, Palmerston North, New Zealand

* Correspondence: Richard.Johnson@agresearch.co.nz

Suppl. Figure S1. Histogram of MAF

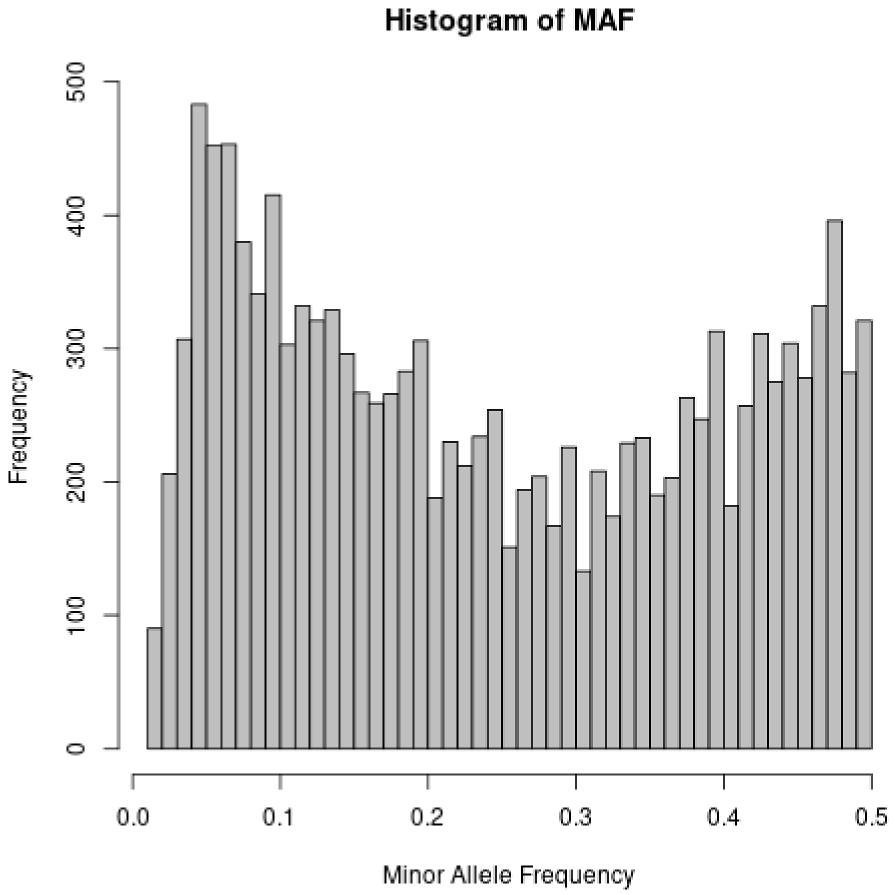

Suppl. Figure S2. Histogram of mean SNP depth

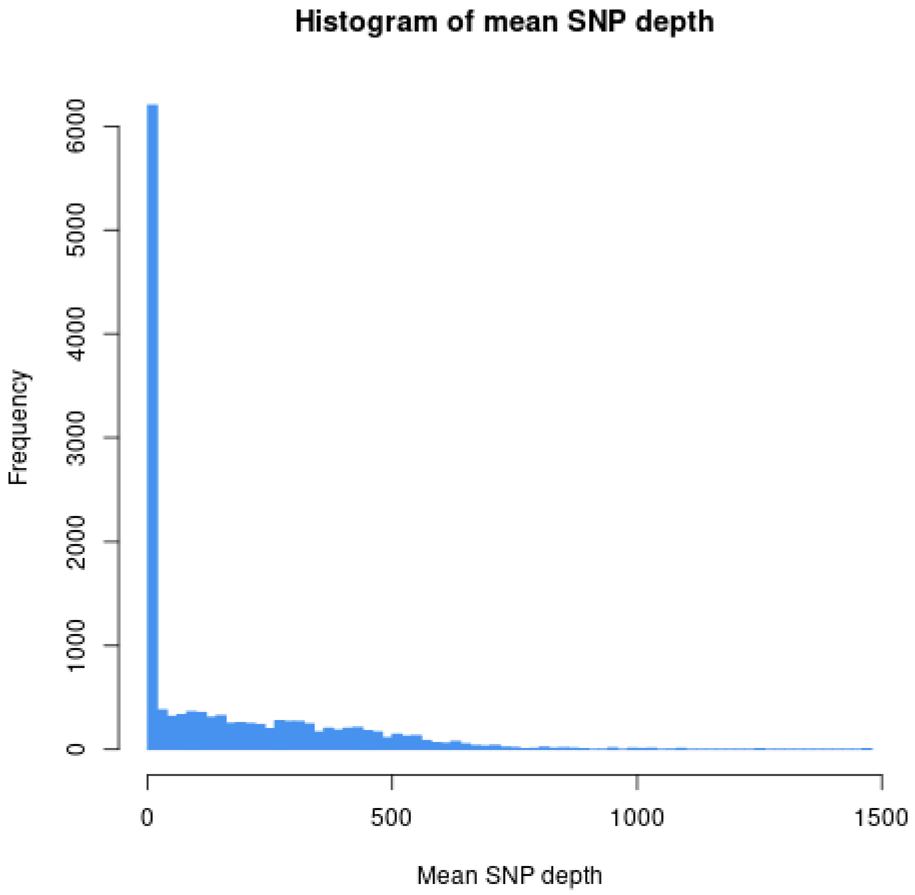

Suppl. Table S1. *Zea* seed accessions screened in this study

Country origin	Host varieties	No. Acc. screened	No. Acc. where *S. zeae* isolated
Mexico	*Zea m. mexicana*	36	19
Mexico	*Zea m. parviglumis*	55	16
Mexico	*Zea m. mays*	46	9
Colombia	*Zea m. mays*	14	4
USA	*Zea m. mays*	40	4
Mexico	*Zea diploperennis*	5	2
Venezuela	*Zea m. mays*	2	1
Barbados	*Zea m. mays*	1	1
Chile	*Zea m. mays*	1	1
Cuba	*Zea m. mays*	1	1
Lebanon	*Zea m. mays*	1	1
Mali	*Zea m. mays*	1	1
Morocco	*Zea m. mays*	1	1
Uruguay	*Zea m. mays*	1	1
Egypt	*Zea m. mays*	2	1
Ethiopia	*Zea m. mays*	2	1
France	*Zea m. mays*	2	1
Poland	*Zea m. mays*	2	1
Soviet Union	*Zea m. mays*	2	1
Thailand	*Zea m. mays*	2	1
Argentina	*Zea m. mays*	3	1
Austria	*Zea m. mays*	3	1
Dominican	*Zea m. mays*	3	1
Japan	*Zea m. mays*	3	1
Brazil	*Zea m. mays*	4	1
China	*Zea m. mays*	5	1
Guatemala	*Zea m. mays*	6	1
Peru	*Zea m. mays*	12	1
Mexico	*Zea perennis*	2	1
Afghanistan	*Zea m. mays*	3	0
Albania	*Zea m. mays*	1	0
Algeria	*Zea m. mays*	2	0
Angola	*Zea m. mays*	2	0
Antigua and Barbuda	*Zea m. mays*	1	0
Australia	*Zea m. mays*	3	0
Azerbaijan	*Zea m. mays*	1	0
Bolivia	*Zea m. mays*	2	0
Botswana	*Zea m. mays*	1	0
Bulgaria	*Zea m. mays*	1	0
Canada	*Zea m. mays*	1	0
Ecuador	*Zea m. mays*	1	0
Eritrea	*Zea m. mays*	1	0

Georgia	*Zea m. mays*	2	0
Germany	*Zea m. mays*	2	0
Hungary	*Zea m. mays*	2	0
India	*Zea m. mays*	7	0
Indonesia	*Zea m. mays*	3	0
Iran	*Zea m. mays*	2	0
Israel	*Zea m. mays*	2	0
Italy	*Zea m. mays*	3	0
Jordan	*Zea m. mays*	1	0
Kazakhstan	*Zea m. mays*	1	0
Kenya	*Zea m. mays*	1	0
Macedonia	*Zea m. mays*	1	0
Moldova	*Zea m. mays*	1	0
Nepal	*Zea m. mays*	1	0
Netherlands	*Zea m. mays*	2	0
New Zealand	*Zea m. mays*	46	0
Nicaragua	*Zea m. mays*	2	0
Nicaragua	*Zea nicarraguensis*	1	0
Pakistan	*Zea m. mays*	2	0
Paraguay	*Zea m. mays*	1	0
Philippines	*Zea m. mays*	3	0
Portugal	*Zea m. mays*	3	0
Romania	*Zea m. mays*	2	0
Serbia and Montenegro	*Zea m. mays*	2	0
South Africa	*Zea m. mays*	3	0
Spain	*Zea m. mays*	2	0
USA	*Zea m. mexicana*	7	0
